# Purification and differentiation of human adipose-derived stem cells by membrane filtration and membrane migration methods

**DOI:** 10.1038/srep40069

**Published:** 2017-01-10

**Authors:** Hong Reng Lin, Chao-Wen Heish, Cheng-Hui Liu, Saradaprasan Muduli, Hsing-Fen Li, Akon Higuchi, S. Suresh Kumar, Abdullah A. Alarfaj, Murugan A. Munusamy, Shih-Tien Hsu, Da-Chung Chen, Giovanni Benelli, Kadarkarai Murugan, Nai-Chen Cheng, Han-Chow Wang, Gwo-Jang Wu

**Affiliations:** 1 Department of Chemical and Materials Engineering, National Central University, No. 300, Jhongda RD., Jhongli, Taoyuan 32001, Taiwan; 2 Department of Botany and Microbiology, King Saud University, Riyadh 11451, Saudi Arabia; 3 Nano Medical Engineering Laboratory, RIKEN, 2-1, Hirosawa, Wako, Saitama 351-0198, Japan; 4 Department of Medical Microbiology and Parasitology, Universiti Putra Malaysia, Serdang 43400, Selangor, Malaysia; 5 Department of Obstetrics and Gynecology, Taiwan Landseed Hospital, 77, Kuangtai Road, Pingjen City, Taoyuan 32405, Taiwan; 6 Department of Internal Medicine, Taiwan Landseed Hospital, 77, Kuangtai Road, Pingjen City, Taoyuan 32405, Taiwan; 7 Department of Agriculture, Food and Environment, University of Pisa, Via del Borghetto 80, 56124 Pisa, Italy; 8 Division of Entomology, Department of Zoology, School of Life Sciences, Bharathiar University, Coimbatore 641 046, Tamil Nadu, India; 9 Department of Zoology, Thiruvalluvar University, Serkkadu, Vellore 632 115, India; 10 Department of Surgery, National Taiwan University Hospital and College of Medicine, 7 Chung-Shan S. Rd., Taipei 100, Taiwan; 11 Hungchi Women & Children’s Hospital, No. 233, Yuanhua Rd., Jhongli, Taoyuan 320, Taiwan; 12 Graduate Institute of Medical Sciences and Department of Obstetrics & Gynecology, Tri-Service General Hospital, National Defense Medical Center, Taipei, Taiwan

## Abstract

Human adipose-derived stem cells (hADSCs) are easily isolated from fat tissue without ethical concerns, but differ in purity, pluripotency, differentiation ability, and stem cell marker expression, depending on the isolation method. We isolated hADSCs from a primary fat tissue solution using: (1) conventional culture, (2) a membrane filtration method, (3) a membrane migration method where the primary cell solution was permeated through membranes, adhered hADSCs were cultured, and hADSCs migrated out from the membranes. Expression of mesenchymal stem cell markers and pluripotency genes, and osteogenic differentiation were compared for hADSCs isolated by different methods using nylon mesh filter membranes with pore sizes ranging from 11 to 80 μm. hADSCs isolated by the membrane migration method had the highest MSC surface marker expression and efficient differentiation into osteoblasts. Osteogenic differentiation ability of hADSCs and MSC surface marker expression were correlated, but osteogenic differentiation ability and pluripotent gene expression were not.

Human adipose-derived stem cells, hADSCs, can be obtained by isolation from fat tissue, which is currently a more practical source of stem cells than human induced pluripotent stem cells (hiPSCs)^1^,^2^,^3^,^4^ and embryonic stem cells (hESCs)^5^. Currently, several clinical trials use hADSCs^6^,^7^,^8^, whereas only a few clinical trials have been performed using hiPSCs and hESCs^9^,^10^,^11^,^12^,^13^. However, hADSCs are known to show heterogeneous characteristics and contain different pluripotency and differentiation abilities. Therefore, it is expected that the stem cell characteristics, pluripotency, and differentiation abilities should be different for hADSCs isolated by different isolation methods. hADSCs are typically isolated by cell culture of stromal vascular fraction (SVF, primary hADSC solution) where the SVF solution can be obtained by collagenase digestion of fat tissues followed by centrifugation (Fig. 1a). Mesenchymal stem cell (MSC) marker expression typically increases after SVF solution is cultured on conventional tissue culture polystyrene (TCPS) dishes^14^,^15^,^16^. MSC surface markers in SVF solution often show less than 10–20% expression, whereas MSC surface markers of the cells after culture on TCPS (i.e. hADSCs) increase to over 80%, which generally indicates that the culture of SVF solution on TCPS dishes leads to the “purification of hADSCs”. Typically, higher expression of MSC surface markers on hADSCs is found with increasing passage number^14^,^17^,^18^,^19^. However, we found that expression of some pluripotent genes such as *Oct3/4, Nanog*, and *Klf4*, which were typical genes used for the transduction of somatic cells in reprogramming into hiPSCs^1^,^2^,^3^,^4^, decreased after culture of cells in SVF solution on TCPS in our previous study^16^, whereas the pluripotent gene expression of the cells in SVF solution could be maintained after purification by filtration via porous membranes^16^. These surprising results indicate that hADSC characteristics depend on the method used for isolation of hADSCs from SVF solution.

There are several possible methods of isolating hADSCs from SVF solution other than the conventional cell culture method on TCPS dishes: (a) MACS (magnetic-activated cell sorting) method^20^, (b) FACS (fluorescence-activated cell sorting) method^21^,^22^, membrane filtration method^15^,^23^,^24^, and membrane migration method^16^. MACS and FACS use antibodies to purify specific target cells such as CD166, CD105, CD90, CD73, CD44, and CD29 expressing cells (CD166^+^, CD105^+^, CD90^+^, CD73^+^, CD44^+^, and CD29^+^ cells). hADSCs isolated by MACS or FACS can be used for research, but not for clinical applications, because antibodies are typically produced using animal-derived materials and make isolation of large quantity of hADSCs extremely expensive. hADSCs as well as human bone marrow stem cells (hBMSCs) are commonly used at a dose of 10^6^–10^7^ cells/kg in clinical applications^7^,^8^,^25^,^26^. Therefore, the membrane filtration method and/or migration method could be an attractive method for the isolation of hADSCs from SVF solution because of short processing time, sterility, and simplicity of the method. In particular, hADSCs can be isolated from SVF solution within 30 min using the membrane filtration method, whereas the conventional culture method typically takes 1–2 weeks to purify hADSCs from SVF solution^15^,^23^,^24^.

In our previous study^15^, hADSCs were purified from SVF solution by the membrane filtration method through polyurethane (PU) foam membranes having 11 μm pore size. SVF solution was permeated through the PU membranes followed by the permeation of the culture medium to detach hADSCs into the culture medium (recovery solution) (Fig. 1a). In this case, the mechanism of isolation of hADSCs through PU membranes is based on the strong affinity of hADSCs to the PU membranes as the affinity membranes. hADSCs were also purified by the membrane filtration method using different type of porous membranes. hADSCs were isolated from SVF solution in permeation solution by filtration through porous silk screen/poly(lactide-co-glycolic acid) membranes using the sieving effect^23^.

In our latest study^16^, we combined the membrane filtration and culture methods, which together were named the membrane migration method. SVF solution was filtered through various porous membranes having 11–25 μm pores, followed by permeation of washing solution (recovery solution). The membranes where hADSCs still adhered, after permeation of SVF solution followed by the washing solution, were cultured on polystyrene dishes (e.g. TCPS or non-treated polystyrene (PS) dishes) in cell culture medium for 14–16 days^16^. Surprisingly, hADSCs migrated out from the membranes^16^. The migrated cells expressed MSC surface markers at higher levels than cells in SVF solution after culture on TCPS or PS dishes (cells purified by the conventional culture method)^16^. However, the effect of pore sizes of the membranes on the pluripotency and purity of hADSCs in the membrane migration method was not investigated in our previous study^16^.

Here, we investigate the pluripotency, purity, and differentiation ability of hADSCs which were isolated by the conventional culture method, membrane filtration method, or the membrane migration method using different pore sizes of nylon (NY) mesh filters (11, 20, 41, 60, and 80 μm). The goal of this study is to find the optimal conditions to purify hADSCs having high pluripotency and differentiation ability using the membrane filtration method and/or membrane migration method, which show better performance than hADSCs purified from SVF solution by the conventional culture method.

## Results

### Permeation of SVF solution through NY mesh filters having different pore sizes

**Figure 1:**
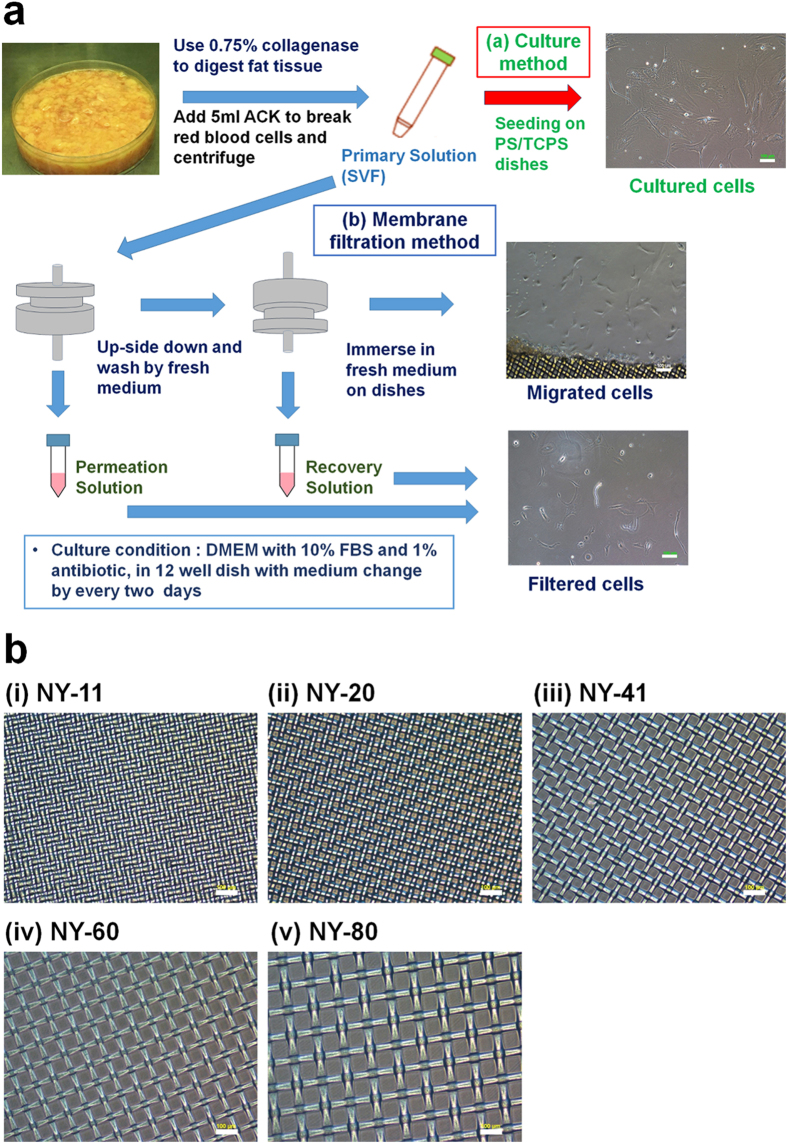
Purification of hADSCs from adipose tissue using the culture, membrane filtration, and membrane migration methods. (**a**) Procedure for the purification of hADSCs. The primary adipose tissue cell solution (SVF) was prepared from fat tissue by collagenase digestion. The SVF solution was cultured on polystyrene (PS) or tissue culture polystyrene (TCPS) dishes to isolate hADSCs by the conventional culture method. In the membrane filtration method, SVF solution was permeated through the membranes and the permeation solution was collected. Subsequently, the membrane holder was inverted and the culture medium was permeated through the membranes where the recovery solution was collected. hADSCs were isolated in the permeation and/or recovery solution in the membrane filtration method. In the membrane migration method, the membranes were cultured on PS dishes in the culture medium after SVF solution and subsequently the culture medium was permeated through the membranes as described above. hADSCs migrated from the membranes on the PS dishes were collected in the migration method. (**b**) Morphology of nylon mesh of NY-11 mesh filter (i), NY-20 mesh filter (ii), NY-41 mesh filter (iii), NY-60 mesh filter (iv), and NY-80 mesh filter (v) used as membranes in membrane filtration and migration methods. The scale bars indicate 100 μm.

**Figure 2:**
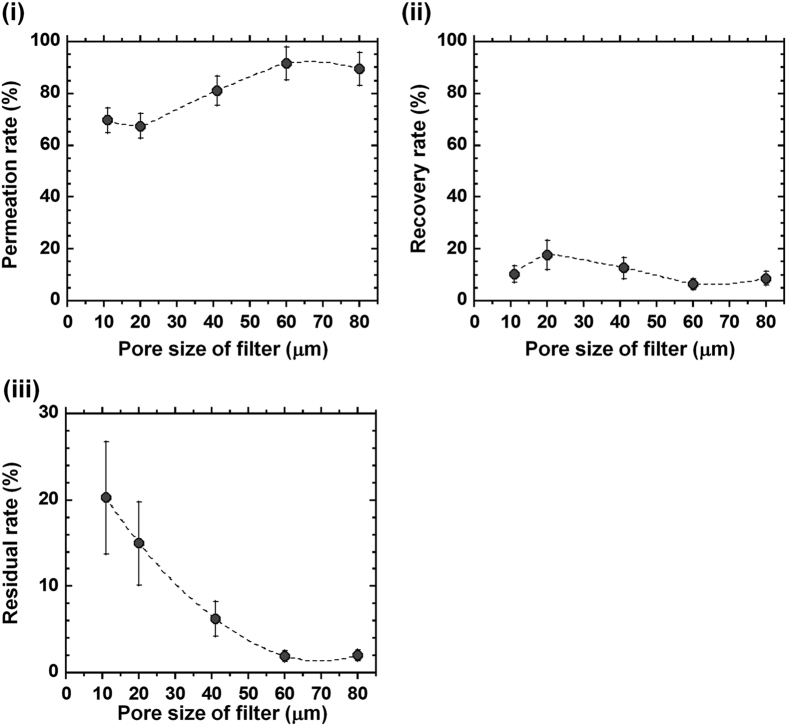
Effect of the pore sizes of the NY mesh filters on permeation, recovery, and residual rate of SVF solution in membrane filtration method. SVF solution was permeated through NY mesh filters with different pore sizes (NY-11, NY-20, NY-41, NY-60, and NY-80 mesh filters). (i) The permeation rate of the cells where SVF solution was permeated through NY mesh filters. (ii) The recovery rate of the cells where SVF solution was permeated and subsequently the culture medium was permeated through NY mesh filters. (iii) The residual rates of the cells, which were calculated from eq. (3).

SVF (primary adipose tissue cell) solution was permeated through NY mesh filters having different pore sizes (NY-11 [11 μm], NY-20 [20 μm], NY-41 [41 μm], NY-60 [60 μm], and NY-80 [80 μm]) for the isolation of hADSCs by the membrane filtration method (Fig. 1a and b). Figure 2 shows the effect of pore sizes of the membranes on the permeation rate and recovery rate as well as the residual rate, which were estimated from the permeation rate and recovery rate (see eq. 3 in Methods). The permeation rate was found to be relatively high (i.e. >65%) compared to the recovery rate (<20%) and residual rate (<25%) in permeation of SVF solution through NY mesh filters having any of the pore sizes in this study. This is because NY mesh filters have relatively simple morphologies, being composed of a single layer of mesh structure (Fig. 1b). Therefore, the cells can easily permeate through NY mesh filters. The permeation rate through NY mesh filters having 11 and 20 μm pore sizes was found to be around 70%, whereas the permeation rate increased with increasing pore size when the pore size of NY mesh filters was in the range of 20–60 μm. This is explained by a more open structure of NY mesh filters obtained with increased pore size of the filters. The residual rate was found to decrease with increasing pore size of NY mesh filters, which is explained by the decrease of surface area per unit mesh area with increase of the pore size of NY mesh filters (Fig. 1b). The residual rate on NY mesh filters having pore size >41 μm was found to be less than 3%. Therefore, we suggest that NY mesh filters having pore size >41 μm should not be used in the membrane migration method, because only a few cells adhered to the NY mesh filters.

The residual cells remaining on the membranes were cultivated on PS dishes in culture medium, and the migrated cells were analyzed for pluripotency and MSC surface marker expression (i.e. purity of hADSCs) in the following sections.

### Culture of migrated cells from NY mesh filters of different pore sizes

**Figure 3:**
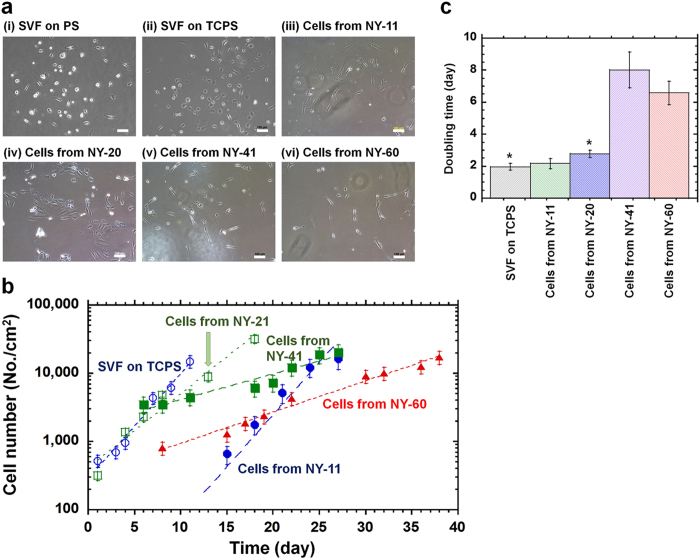
Cultivation of hADSCs isolated from the conventional culture and membrane migration methods. (**a**) The morphology of the cells isolated by the culture method from SVF solution on PS dishes (i) and TCPS dishes (ii) and the morphology of the cells migrated from NY-11 (iii), NY-20 (iv), NY-41 (v), and NY-60 (vi) filters using the membrane migration method. (**b**) Growth curve of the cells isolated by the culture method from SVF solution on TCPS dishes (blue open circle) and the cells migrated from NY-11 (blue closed circle), NY-21 (green open square), NY-41 (green closed square), and NY-60 (red closed triangle) mesh filters using the membrane migration method. (**c**) Doubling time of the cells isolated from conventional culture method where SVF solution was cultured for 11 days on TCPS dishes (SVF on TCPS) and doubling time of the cells migrated from NY-11 (Cells from NY-11), NY-21 (Cells from NY-21), NY-41 (Cells from NY-41), and NY-60 (Cells from NY-60) mesh filters using the membrane migration method. * indicates statistical significance (*p* < 0.05).

The migrated cells from NY mesh filters having different pore sizes were cultured on PS dishes in DMEM with 12% fetal bovine serum (FBS). The reason why the adhered cells migrated from NY mesh filters is explained by the migration characteristics of hADSCs. The adhered cells on the filters tended to migrate from areas of high cell density into open spaces of cell culture dishes that were located in fresh and high nutrition regions. Figure 3a shows the morphologies of (1) the migrated cells from NY mesh filters having different pore sizes where hADSCs were isolated from SVF solution by the membrane migration method and (2) the cells in SVF solution cultured on TCPS and PS dishes where hADSCs were purified by conventional culture method. Most of the cells migrated from NY mesh filters having any pore size showed spindle shape morphology Fig. 3a(iii, iv, v and vi), whereas a variety of cell morphologies were found for the cells purified from the conventional culture method Fig. 3a(i and ii).

Cell growth curves are shown in Fig. 3b for (a) cells in SVF solution cultured on TCPS dishes and (b) the migrated cells from NY mesh filters having different pore sizes that were cultured on PS dishes. The doubling times of these cells calculated from Fig. 3b are summarized in Fig. 3c. The doubling time of cells in SVF solution cultured on TCPS dishes by the conventional culture method appeared to be approximately 2.0 days, which is similar to the doubling time reported by several investigators^14^,^17^,^27^. The doubling time of the migrated cells from NY mesh filters having a pore size of 11 μm showed similar doubling time, whereas the doubling time of the migrated cells from the filter having pore size >20 μm that were cultured on PS dishes was longer than the doubling time of the cells in SVF solution cultured on TCPS dishes (*p* < 0.05). In particular, the doubling time of the migrated cells from NY mesh filters having 41 and 60 μm pore sizes that were cultured on PS dishes was found to be 6.5–8.0 days, which was more than 2–3 fold longer than the doubling time of the cells in SVF solution cultured on TCPS dishes and the migrated cells from NY mesh filters having 11 and 20 μm pore sizes that were cultured on PS dishes. This is probably because smaller numbers of hADSCs remained on the NY mesh filters having 41 and 60 μm pore size than those on the filters having 11 and 20 μm pore sizes. This explanation is supported by the extremely low value of residual rate when SVF solution and subsequently washing solution were permeated through NY mesh filters having 41 and 60 μm pore sizes compared to the residual rate for NY mesh filters having 11 and 20 μm pore sizes Fig. 2(iii). We could not get reliable data of migrated cells from 80 μm pore size NY mesh filters.

Because there is no commercially available NY mesh filter having a smaller pore size than 10 μm, we suggest that NY mesh filters with pore sizes ranging from 11 μm–20 μm are the preferred filters for the isolation of hADSCs by the membrane migration method.

### Surface marker expression of hADSCs purified by the culture method, membrane filtration method, or membrane migration method

The characteristics of hADSCs purified by the (a) conventional culture method, (b) filtration method, and (c) migration method were evaluated for MSC surface marker expression by flow cytometry and for pluripotent gene expression by qRT-PCR.

**Figure 4:**
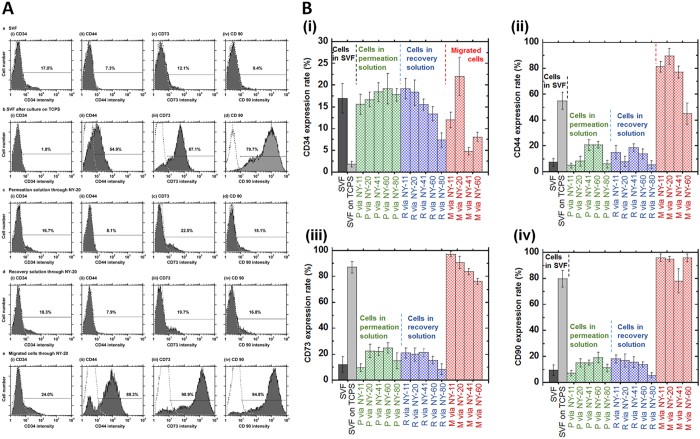
CD34 and MSC surface marker expression of hADSCs isolated using the conventional culture, membrane filtration, and membrane migration methods. (**A**) Flow cytometry analysis of the expression of CD34 (i) and MSC surface markers (CD44 [ii], CD73 [iii], and CD90 [iv]) on cells in SVF solution (SVF, the primary adipose tissue cells) (a), cells isolated by the culture method on TCPS dishes at first passage (SVF after culture on TCPS) (b), cells in permeation solution by the membrane filtration method through NY-20 mesh filters (Permeation solution through NY-20) (c), cells in recovery solution by the membrane filtration method through NY-20 mesh filters (Recovery solution through NY-20) (d), and cells that migrated out from NY-20 mesh filters that were subsequently cultured on PS dishes for 18 days after SVF solution and subsequently culture medium was permeated through the filters (Migrated cells through NY-20) (e). The dotted lines represent the cells stained with the isotype antibody as negative controls. (**B**) As analyzed by flow cytometry, the expression of CD34 (i), CD44 (ii), CD73 (iii) and CD90 (iv) on cells in SVF solution (SVF), cells isolated by the culture method on TCPS dishes at first passage, (SVF on TCPS), cells in permeation solution by the membrane filtration method through NY-11 (P via NY-11), NY-20 (P via NY-20), NY-41 (P via NY-41), NY-60 (P via NY-60), and NY-80 (P via NY-80) mesh filters, cells in recovery solution by the membrane filtration method through NY-11 (R via NY-11), NY-20 (R via NY-20), NY-41 (R via NY-41), NY-60 (R via NY-60), and NY-80 (R via NY-80) mesh filters, and cells that migrated out from NY-11 (M via NY-11), NY-20 (M via NY-20), NY-41 (M via NY-41), NY-60 (M via NY-60) mesh filters that were subsequently cultured on PS dishes.

Figure 4A presents representative results of flow cytometry for the expression of CD90, CD73, CD44, and CD34 for (i) cells in SVF solution (primary adipose tissue cells) Fig. 4A(a), (ii) cells isolated by the culture method (the first-passage cells in SVF solution on TCPS) Fig. 4A(b), (iii) the cells in permeation solution through NY mesh filter (NY-20) Fig. 4A(c), and (iv) the cells in recovery solution through NY mesh filter (NY-20) Fig. 4A(d), and the cells that migrated from the NY mesh filter (NY-20) Fig. 4A(e). The cells in SVF solution showed low expression (less than 15%) of MSC surface markers (CD90, CD73, and CD44) Fig. 4A(b), whereas the expression of MSC surface marker of the cells increased after the culture of cells in SVF solution on TCPS dishes Fig. 4A(b), This is similar to results reported by previous researchers^14^,^15^,^16^. The culture method could isolate and expand the cells expressing MSC surface marker in SVF solution. However, it should be mentioned that CD34 expression of the cells in SVF solution decreased after the purification using the cell culture method Fig. 4A(b) where CD34 in hADSCs is considered to be a marker for endothelial progenitor cells and not hematopoietic stem cells^16^. The MSC surface marker expression of the cells in the recovery solution and permeation solution was found to be the same (*p* > 0.05) to that in SVF solution Fig. 4A(a, c, and d). CD34 expression of the cells in the permeation and recovery solution remained similar (*p* > 0.05) to the expression level in SVF solution Fig. 4A(a, c, and d).

The migrated cells from NY-20 mesh filters showed equal or higher expression of MSC surface markers compared to the cells isolated by the conventional culture method (the cells cultured on TCPS) Fig. 4A(b and e). CD34 expression of the migrated cells from NY-20 mesh filters was much higher than that of the cells isolated by the conventional culture method (*p* < 0.05). This is one of the advantages of isolating hADSCs from SVF solution using the migration method compared to hADSCs isolated by the conventional culture method.

Figure 4B displays the expression of CD90, CD73, CD44, and CD34 on the cells in permeation and recovery solution through NY mesh filters having different pore sizes (NY-11, NY-41, NY-60) and for cells migrated from NY mesh filters of different pore sizes (NY-11, NY-41, NY-60). The migrated cells from NY-11, NY-20, NY-41 and NY-60 mesh filters showed higher MSC surface marker expression than the cells in permeation solution and recovery solution from NY mesh filters having pore sizes 11–80 μm (*p* < 0.05). The migrated cells from NY-11 and MY-20 mesh filters in particular gave higher CD44 and CD90 expression (*p* < 0.05) than the cells isolated by the conventional culture method at passage 1.

There was no significant effect of pore sizes on the expression of MSC surface markers on the cells in the permeation solution as well as in the recovery solution, when NY mesh filters having pore sizes between 11 μm and 60 μm were used, whereas CD73, CD44, and CD34 expression of the migrated cells through NY-11 and NY-20 mesh filters was higher than that of migrated cells through NY-60 mesh filters (*p* < 0.05). The migrated cells from NY-11 and NY-20 mesh filters showed CD34 expression similar to that of the cells in SVF solution (*p* > 0.05), whereas CD34 expression of the migrated cells through NY-41 and NY-60 mesh filters was less than that of the cells in SVF solution (*p* < 0.05). Furthermore, the migrated cells from NY-11 and NY-20 mesh filters expressed similar or higher MSC surface markers as well as CD34 surface marker compared to the cells purified from SVF solution by the conventional culture method. The migrated cells from NY-20 mesh filters could be obtained relatively faster than the migrated cells from NY-11 mesh filters (Fig. 3b). Therefore, the osteogenic differentiation ability was evaluated for hADSCs purified by several methods, including permeation and recovery solution through NY-20 mesh filters and migration from NY-20 mesh filters, in the following section.

### Pluripotent gene evaluation of hADSCs isolated by the culture, membrane filtration, and membrane migration methods

**Figure 5:**
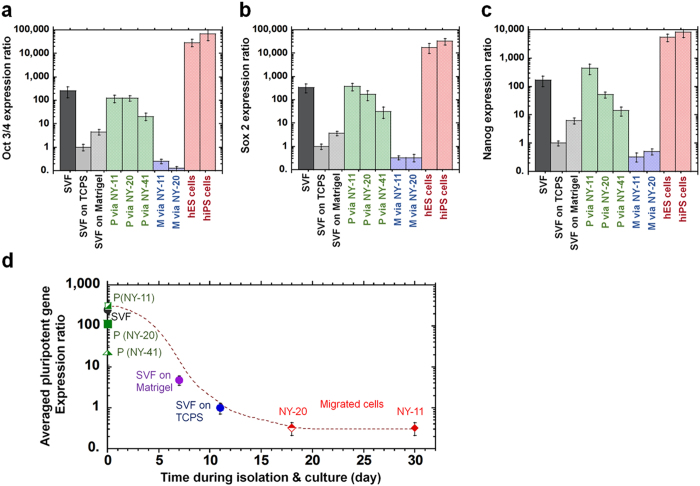
Pluripotency of hADSCs isolated using the conventional culture, membrane filtration, and membrane migration methods. (**a–c**) Relative gene expression levels of *Oct3/4* (**a**), *Sox2* (**b**), and *Nanog* (**c**) as analyzed by qRT-PCR in (i) cells in SVF solution (SVF), cells isolated by the culture method on TCPS dishes at first passage (SVF on TCPS), (ii) cells isolated by the culture method on Matrigel-coated dishes at first passage (SVF on Matrigel), (iii) cells in permeation solution by the membrane filtration method through NY-11 (P via NY-11), NY-20 (P via NY-20), and NY-41 (P via NY-41) mesh filters, and (iv) cells that migrated out from NY-11 (M via NY-11) and NY-20 (M via NY-20) mesh filters and were subsequently cultured on PS dishes as well as those of human ES cells (H9) and human iPS cells (HS0077) as positive controls. (**d**) The dependence of averaged pluripotent gene expression (*Nanog, Sox2*, and *Oct3/4*) of the cells on the cell culturing time where the cells were isolated by the conventional culture, membrane filtration, or membrane migration method. Cells in SVF solution were included for comparison.

Based on the expression of MSC surface markers on hADSCs purified by several methods, we expected that hADSCs isolated using the conventional culture and membrane migration methods, but not by the membrane filtration method, would show a higher pluripotent gene expression than the cells in SVF solution because of higher MSC surface marker expression. Therefore, the expression of the pluripotent genes *Nanog, Sox2*, and *Oct3/4* was investigated by qRT-PCR in (i) the cells in SVF solution, (ii) hADSC cells isolated by the conventional culture method on TCPS dishes, (iii) the cells in permeation solution through NY-11, NY-20, and NY-41 filters, (iv) the migrated cells (hADSCs) from SVF solution through NY-11 and NY-20 mesh filters, and (v) hiPSCs (HS0077) and hESCs (WA09) as positive controls Fig. 5(a–c). Because relatively large number of cells were required to evaluate gene expression by qRT-PCR, it was difficult to evaluate the pluripotent gene expression of the migrated cells from NY mesh filter having pore size >41 μm and the cells in the recovery solution through NY mesh filters having any pore size in this study. Therefore, only the migrated cells from NY-11 and NY-20 mesh filters and the cells in permeation solution through NY-11, NY-20, and NY-41 mesh filters were analyzed here.

The expression of pluripotent genes of hiPSCs and hESCs was found to be 3 to 5 fold higher than that of hADSCs isolated by the conventional culture method on TCPS dishes Fig. 5(a–c). The cells in permeation through NY-11, NY-20, and NY-41 mesh filters expressed higher pluripotent genes of *Nanog, Sox2*, and *Oct3/4* than hADSCs isolated by the conventional culture method on TCPS dishes and Matrigel-coated dishes, and showed similar expression levels of the pluripotent genes to the cells in SVF solution. The migrated cells from NY-11 and NY-20 showed less expression of pluripotent genes compared to the cells in SVF solution, hADSCs isolated by the conventional culture method, and the permeation cells via NY-11, NY-20, and NY-41 mesh filters.

In the previous section, MSC surface marker expression of cells showed the following order:


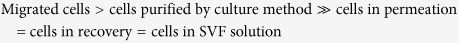


On the other hand, pluripotent gene expression gave the following order:


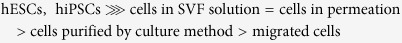


The above relationships clearly indicate that the cells strongly expressing high MSC surface markers do not express pluripotent genes at high levels. Especially, MSCs are known to be purified from SVF solution by the culture method^14^,^15^,^16^, which was verified by increased MSC surface marker expression of the cells after cultivation compared to the cells in SVF solution. However, the cells after cultivation showed a dramatically decreased pluripotent gene expression compared to the cells in SVF solution. The cells in permeation solution could maintain a level of pluripotent gene expression similar to that of the cells in SVF solution, although MSC surface marker expression of the cells in the permeation solution did not improve extensively compared to that of the cells in SVF solution.

The effect of cell culturing time on pluripotent gene expression for cells isolated by different methods is shown in Fig. 5d. Pluripotent gene expression was analyzed by the average expression ratio of *Nanog, Sox2*, and *Oct3/4*. The average pluripotent gene expression decreased with increased culturing time of the cells. This effect will be discussed in more detail in the Discussion.

### Osteogenic differentiation of hADSCs purified by the culture, membrane filtration, or migration methods

hADSCs were isolated from SVF solution by several methods (cells in permeation or recovery solution by membrane filtration method, migrated cells by membrane migration method, and cells by conventional culture method) and were analyzed for expression of MSC surface markers and pluripotent genes as previously. One of the important characteristics of MSCs is the ability to differentiate into cells of the mesoderm lineages. Therefore, the differentiation ability of hADSCs into osteoblasts was tested to investigate the purity of hADSCs isolated by several methods in this study. NY-20 mesh filters were used as the representative filters for the membrane filtration method and the membrane migration method, based on relatively higher expression of MSC surface markers and pluripotency genes as well as faster hADSC isolation compared to the membrane migration method from NY-11, NY-41, NY-60, and NY-80 mesh filters.

**Figure 6:**
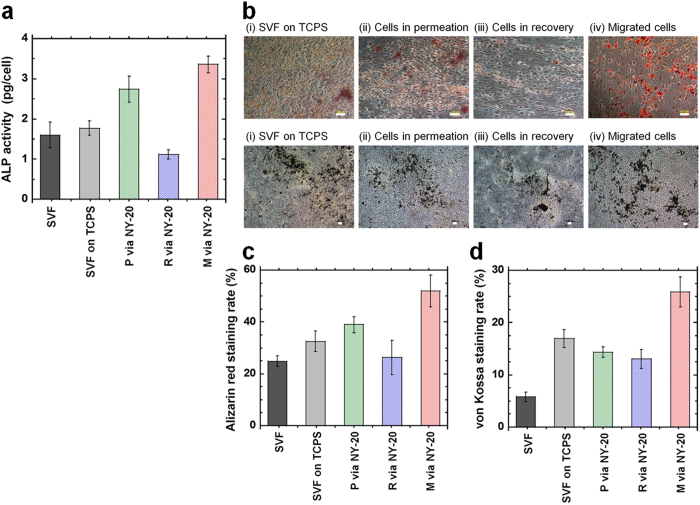
Osteogenic differentiation of hADSCs isolated using the conventional culture, membrane filtration, and membrane migration methods. (**a**) The ALP activity of the cells after osteogenic induction of cells in SVF solution (SVF), cells isolated by the culture method on TCPS dishes at first passage (SVF on TCPS), cells in permeation solution by the membrane filtration method through NY-20 mesh filters (P via NY-20), cells in recovery solution by the membrane filtration method through NY-21 mesh filters (R via NY-21), and cells that migrated out from NY-21 mesh filters and were subsequently cultured on PS dishes (M via NY-21) were cultured for 18 days. (**b**) Micrograph images of cells analyzed by Alizarin Red S staining (i–iv) and von Kossa staining (v–viii) after osteogenic differentiation of the cells that were isolated by the different methods. The cells were cultured for 28 days in osteogenic differentiation media. The scale bar represents 100 μm. (**c**) The level of osteogenic differentiation of cells analyzed by Alizarin Red S staining (calcium deposition) using Image J software after osteogenic differentiation for 28 days of the cells that were isolated by several methods. (**d**) The level of osteogenic differentiation of the cells analyzed by von Kossa staining (calcium phosphate deposition) using Image J software after culturing the cells that were isolated by several methods for 28 days in osteogenic differentiation media.

The expression of the early marker alkaline phosphatase (ALP) of osteogenic cells was evaluated at day 14, following induction of osteogenic differentiation for the cells in SVF solution and hADSCs purified by several methods (Fig. 6a). The ALP activity of the cells differentiated from the cells in SVF solution and hADSCs purified from SVF solution using the conventional culture method on TCPS dishes showed almost the same level (*p* > 0.05), whereas the ALP activity of the differentiated cells from the cells in recovery solution through NY-20 mesh filters was found to be slightly lesser than that of the cells in SVF solution (*p* < 0.05). The differentiated cells from the cells in permeation solution and the migrated cells from NY-20 mesh filters represented higher ALP activity than the cells differentiated from the cells in SVF solution, the cells isolated by the culture method, and the cells in recovery solution through NY-20 mesh filters (*p* < 0.05). The differentiated cells from the migrated cells from NY-20 mesh filters showed the highest ALP activity among the differentiated cells investigated in this case (*p* < 0.05).

The index marker of late osteogenic differentiation, mineralization, was investigated by von Kossa staining and Alizarin Red S staining. Mineralization was assessed for cells differentiated from the cells in SVF solution and from hADSCs isolated by several methods (membrane filtration, membrane migration, and conventional culture). The images of cells stained with von Kossa and Alizarin Red S are shown in Fig. 6b. The rate of the stained cells was analyzed using ImageJ software (NIH, https://imagej.nih.gov/ij/) and the results for Alizarin Red S staining are summarized in Fig. 6c and those for are summarized in von Kossa staining Fig. 6d. The cells differentiated from the cells in SVF solution showed minimal staining for both von Kossa and Alizarin Red S staining in this study (*p* < 0.05), whereas the differentiated cells from the migrated cells from NY-20 mesh filters represented the highest staining rate (*p* < 0.05). The differentiated cells from the cells isolated by the culture method and the cells in permeation cells through NY-20 mesh filters showed a higher staining rate for both von Kossa and Alizarin Red S staining than the differentiated cells from the cells in SVF solution (*p* < 0.05). The differentiated cells, which were derived from the recovery solution through NY-20 mesh filters, stained at similar levels to differentiated cells, which were obtained via the culture method and the permeation method through NY-20 mesh filters.

## Discussion

MSCs isolated from human tissues, such as hADSCs, are known to possess heterogeneous characteristics, including various genotypes and differentiation abilities. hADSCs purified by different methods have different levels of stemness and purity. Cells isolated by the membrane filtration, membrane migration, and conventional culture methods were compared. The membrane filtration method took less than 30–40 min to isolate cells from SVF solution in permeation or recovery solution. In contrast, isolation of hADSCs using the conventional culture method took 1–2 weeks. The membrane migration method needed 2–4 weeks for the isolation of hADSCs; however, hADSCs isolated by the membrane migration method showed the highest MSC surface marker expression (Fig. 4b) and the highest osteogenic differentiation ability (Fig. 6c and d) among the hADSCs isolated by different methods in this study (*p* < 0.05). Isolating cells in permeation solution from SVF solution through NY-20 (and NY-11) mesh filters should be the desirable purification method for the rapid isolation of hADSCs based on the results of higher osteogenic differentiation index than the cells in SVF solution and osteogenic differentiation ability similar to that of the cells isolated by the conventional culture method (Fig. 6c and d). Cells migrated from NY-20 mesh filters showed the highest MSC surface marker expression as well as the highest osteogenic differentiation ability. Therefore, isolation by the membrane migration method gave the highest purity of hADSCs, although it took 2–4 weeks for the isolation of hADSCs by this route.

hADSCs purified by culturing cells on TCPS dishes in the conventional culture method showed dramatically reduced pluripotent gene expression compared to the cells in SVF solution. In contrast, MSC surface marker expression of cells isolated by culturing on TCPS dishes was enhanced compared to that of the cells in SVF solution. One factor determining the level of pluripotent gene expression may be the culture period for cells in SVF solution. The average pluripotent gene expression decreased with increase in culturing time (Fig. 5d). This result indicates that SVF solution should contain the cells having high pluripotent gene expression. However, it is difficult to maintain specific cells expressing pluripotent genes at high levels in culture. The number of cells with a high level of pluripotent gene expression decreases during culture. Other researchers have noted decreasing pluripotent gene expression of hADSCs over time during monolayer culture^28^. Park and Patel reported that the pluripotent gene expression of *Nanog, Oct-4,* and *Rex-1* in hADSCs cultured on TCPS dishes decreased with increasing passage number^28^. The average pluripotent gene expression for the cells in SVF solution cultured on Matrigel-coated plates was higher than that for cells cultured on TCPS plates (Fig. 5) (p < 0.05). However, this result seems to be because of faster cell growth on Matrigel-coated dishes compared to TCPS dishes. Currently, there is no optimal culturing method to maintain hADSC pluripotency during cultivation. One method to keep MSC pluripotency during cultivation may be suspension culture, which is similar to the environment of the cells in SVF solution.

Cheng *et al*. investigated whether enhanced pluripotent gene expression could be achieved by culturing hADSCs on TCPS dishes in two-dimensional (2D) monolayer culture following three-dimensional (3D) spheroid culture^29^,^30^. hADSCs after 3D culture kept the expression pattern of MSC surface markers as well as osteogenic and adipogenic differentiation abilities. In particular, hADSCs after 3D culture exhibited higher pluripotent gene expression and expansion efficiency with less senescence^29^,^30^.

Several researchers reported that the formation of hADSC spheroids enhanced expression of pluripotent proteins and genes compared to 2D monolayer culture^31^,^32^,^33^,^34^. Mineda *et al*. found by microarray experiments that hADSC spheroids in hyaluronic acid gels upregulated several pluripotent genes (*Nanog, Oct3/4*, and *STAT3*) compared to hADSCs in monolayer culture^31^. Furthermore, they found by immunostaining of hADSCs that 40% of the spheroids expressed pluripotent proteins of SSEA-4, whereas only 1% of hADSCs in monolayer culture expressed SSEA-4^31^.

Another possible method to enhance hADSC pluripotency is culture in the presence of specific molecules such as L-ascorbate 2-phosphate, which is a stable derivative of ascorbic acid^35^, or in optimal culture medium^17^. Yu *et al*. reported that hADSCs cultured in medium with added L-ascorbate 2-phosphate could proliferate extensively and showed enhanced expression of the pluripotent markers *Nanog, Sox-2*, and *Oct-4*^35^.

**Figure 7:**
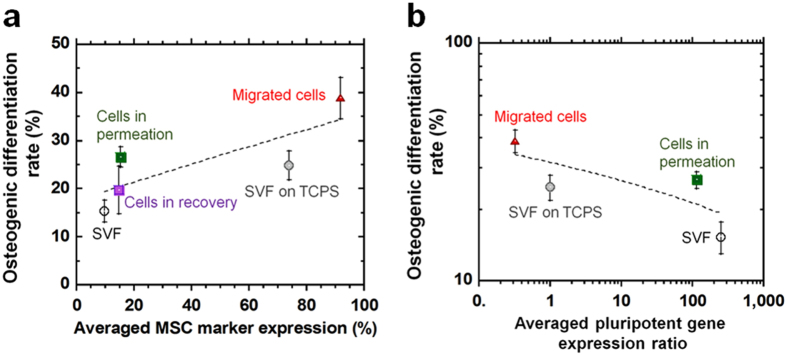
The relationship between osteogenic differentiation ability and MSC surface marker expression or pluripotent gene expression of hADSCs, which were purified from different isolation methods from SVF solution. (**a**) The relationship between averaged MSC marker expression and averaged osteogenic differentiation rate of the cells, which were differentiated from (i) cells in SVF solution (SVF), (ii) cells isolated by membrane filtration method in permeation solution through NY-20 mesh filters (Cells in permeation), (iii) cells isolated by membrane filtration method in recovery solution through NY-20 mesh filters (Cells in recovery), (iv) migrated cells isolated by membrane migration from NY-20 mesh filters that were subsequently cultured on PS dishes for 18 days (Migrated cells), and (v) cells by culture method, which were cultured on TCPS dishes for 7 days (SVF on TCPS). (**b**) The relationship between averaged MSC marker expression and averaged pluripotent gene expression ratio of the cells, which were differentiated from (i) cells in SVF solution (SVF), (ii) cells isolated by membrane filtration method in permeation solution through NY-20 mesh filters (Cells in permeation), (iii) Cells by membrane migration from NY-20 mesh filters that were subsequently cultured on PS dishes for 18 days (Migrated cells), and (iv) cells by culturing on TCPS dishes for 7 days (SVF on TCPS).

It is an interesting question whether differentiation ability of hADSCs is related to the pluripotency of the cells (high pluripotent gene expression) or the characteristics of MSCs (high MSC surface marker expression). The relationship between average MSC surface marker expression and averaged osteogenic differentiation rate was investigated following induction of differentiation (Fig. 7a). Cells in SVF solution were compared with cells isolated by the different methods (membrane filtration, membrane migration, and conventional culture). The average MSC surface marker expression was calculated from the averaged MSC surface marker expression rate of CD90, CD73, and CD44, whereas the average osteogenic differentiation rate was evaluated from the average rate of Alizarin Red S and von Kossa stained cells. Furthermore, the relationship between average pluripotent gene expression (from expression ratios of *Nanog, Sox2*, and *Oct3/4*) and average osteogenic differentiation rate of the cells was evaluated (Fig. 7b). The osteogenic differentiation rate of the cells increased with increasing MSC surface marker expression (Fig. 7a), whereas the osteogenic differentiation rate of the cells decreased with increasing pluripotent gene expression (Fig. 7b). However, only four points are shown in the plot of average osteogenic differentiation rate of the cells vs. average pluripotent gene expression ratio of the cells (Fig. 7b), because pluripotent gene expression for the cells in recovery solution could not be evaluated owing to a shortage in cell numbers. Therefore, the average osteogenic differentiation rate of the cells was found to be more related to the averaged MSC surface marker expression of the cells (correlation coefficient *r*^2^ = 0.633) than the averaged pluripotent gene expression ratio. These results indicate that the pluripotency of hADSCs does not directly relate to the ability to differentiate into osteoblasts but the stemness of hADSCs, as measured by high expression of MSC surface markers, is more important for the differentiation of hADSCs into osteoblasts. However, there is a possibility that this relationship between average MSC surface marker expression and hADSC differentiation ability might only be valid for differentiation into osteoblasts. Therefore, work should be done in the future to evaluate the ability of hADSCs to differentiate into other cell lineages, such as chondrogenic and adipogenic cells (mesodermal cells) as well as neural cells, which are not from the mesodermal lineage but are ectodermal cells (trans-differentiation). These results would be of great value in the further study of hADSCs isolated by the different methods used here.

## Methods

### Preparation of SVF solution

The experiments in this study were approved by the ethics committees of the Taiwan Landseed Hospital (IRB-13–05), the National Central University, and the Cathay Medical Research Institute (CT099012). All experiments were done in accordance with all relevant and applicable governmental and institutional guidelines and regulations. Human adipose tissue was collected from the fat pads of human patients from the omentum and near the intestines (45–85 years old, six people) after obtaining informed consent. The adipose tissue cell solution (SVF, stromal vascular fraction) was made using a previously reported method^16^,^23^,^36^. The number of hADSCs in the adipose tissue cell solution was evaluated using flow cytometry with antibodies to CD90, CD73, CD40, and CD34 and appropriate isotype antibodies. The total cell number in the solution was also evaluated by flow cytometry (Coulter EPICS™ XL, Beckman Coulter, Inc., Marseille, France) after staining with 7-AAD (A07704, Beckman Coulter, Inc., Marseille, France). hADSCs were purified from SVF solution using (a) the conventional culture method^16^,^36^,^37^, (b) the filtration method through Nylon (NY) mesh filters with pore sizes of 11, 20, 41, 60, and 80 μm (NY-11, NY-20, NY-41, NY-60, and NY-80, respectively), and (c) the migration method using NY-11, NY-20, NY-41, NY-60, and NY-80 mesh filters.

### Purification of hADSCs using the culture method

The conventional culture method was used to purify hADSCs from SVF solution. The resulting hADSCs were compared for purity with the hADSCs purified by the membrane filtration method^15^,^23^ and by the membrane migration method^16^. The cells in the SVF solution were inoculated on untreated PS dishes (351029, Falcon^R^, BD Biosciences, USA) or TCPS (430167, Corning Incorporated, USA) dishes at 10^4^ cells/cm^2^ in DMEM containing 12% FBS at 37.0 °C in a 5.0% CO_2_ incubator. When the cells reached approximately 85–90% confluence (8–11 days), the cells were passaged using a conventional cell passage method. The hADSCs thus prepared were resuspended in DMEM containing 12% FBS and were utilized for analysis^15^,^16^,^23^.

### hADSCs purification using the membrane filtration method

hADSCs were isolated from SVF solution by membrane filtration through NY mesh filters having a pore size of 11, 20, 41, 60, or 80 μm (Fig. 1). Similar membrane holders and permeation methods to those reported previously^23^ were used in this study (Fig. 1a). SVF solution (feed solution) with a total number of 1 × 10^6^ cells in 18 mL was filtered through NY mesh filters at 25.0 °C (Fig. 1a) with a permeation rate of 1–1.2 mL/min. The numbers of hADSCs in the permeation solution and the feed solution were evaluated using flow cytometry with antibodies against CD90, CD73, CD40, and CD34, and their isotype controls. The total cell numbers in the feed solution and the permeate solution (*N*_f_ and *N*_p_, respectively) were evaluated using flow cytometry.

Following permeation, the membrane holder was inverted, and DMEM containing 12% FBS (recovery solution) was filtered through the NY mesh filters at a permeation speed of 1–1.2 mL/min at 25.0 °C. This step was done to collect the cells that were adhered to the NY mesh filters in the recovery solution (Fig. 1a). The number of hADSCs in the recovery solution was evaluated using flow cytometry with antibodies against CD90, CD73, CD40, and CD34, and their isotype controls. The total cell number in the recovery solution (*N*_r_) was evaluated using flow cytometry.

The permeation rate was described as


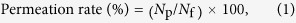


the recovery rate was described as


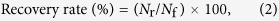


and the residual rate was described as


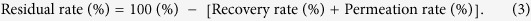


### hADSCs purification using the membrane migration method

hADSCs were also isolated from SVF solution using migration from membranes^16^. The membranes used in the membrane filtration method after filtration of the recovery solution (washing solution) were subsequently detached from the membrane holder and inputted into PS dishes containing DMEM containing 12% FBS. The residual cells adhered on the NY mesh filters began to migrate out from the NY mesh filters into the cell culture plates, and were incubated for 7–38 days at 37.0 °C in a 5.0% CO_2_ incubator. The number of the migrated cells (hADSCs) was evaluated using flow cytometry with antibodies against CD90, CD73, CD40, and CD34, and their isotypes as control experiments. The total cell number in the cell culture medium was determined by flow cytometry.

### hESC and hiPSC culture

Human iPS cell line, HPS0077, was received from Riken Cell Bank (Tsukuba, Japan). Human ES cell line WA09 (H9) was purchased from the WiCell Research Institute (Madison, WI). Human iPS and ES cells were cultivated on Matrigel-coated dishes in the chemically defined medium, Essential 8 (A1517001, Thermo Fisher Scientific Inc., Waltham, Massachusetts, USA) at 37.0 °C and 5.0% CO_2_ with daily medium exchange using the conventional culture method for human ES and iPS cells^38^.

### Pluripotency assay of hADSCs

The expression levels of the pluripotent genes *Nanog, Sox2,* and *Oct4* were evaluated by qRT-PCR using conventional methods^39^,^40^. Probes for *GAPDH* (glyceraldehyde-3-phosphate dehydrogenase, Hs03929097_g1), *Nanog* (Hs02387400_g1), *Sox2* (Hs00602736_s1), and *Oct4* (Hs01895061_u1) were received from Life Technologies (Carlsbad, California). Each sample (*n* = 3) was evaluated in duplicate, and the expression level of the GAPDH housekeeping gene was utilized as a control to normalize the results^41^.

### Osteogenic differentiation of hADSCs

hADSCs (2–3 × 10^4^ cells) were inoculated in DMEM containing 12% FBS and cultivated for 24 h before the medium was changed to an induction medium for osteoblasts. hADSCs were cultivated in the osteogenic medium (CCM007, R&D Systems, Inc., USA) with a supplement (CCM009, R&D Systems, Inc., USA) for up to 28–30 days. The medium was exchanged twice weekly^23^.

The alkaline phosphate activity, ALP, of the osteogenic differentiated cells was investigated after 14 days of osteogenic differentiation period using the Alkaline Phosphatase Assay Kit (72146, AnaSpec, San Jose, CA) according to the manufacturer’s protocol^42^.

The cells were treated with von Kossa (S7276; Sigma-Aldrich) staining to evaluate calcium phosphate and Alizarin Red S (A5533; Sigma-Aldrich) staining to evaluate calcium, both of which are markers of osteogenic differentiation^15^,^23^,^24^. Micrographs of the stained cells were taken with an inverted phase microscope (Eclipse Ti, Nikon Co., Tokyo, Japan). The images were investigated using ImageJ software (http://rsb.info.nih.gov/ij/) to obtain the numbers of unstained and stained cells.

The osteogenic differentiation rate was evaluated as


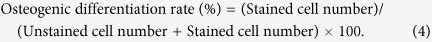


### Statistical analysis

All of the quantitative results were obtained from four samples. The data are shown as the mean ± SD. Statistical analyses were evaluated from the unpaired Student’s *t*-test in Excel (Microsoft Corporation). Probability values (*p*) less than 0.05 were regarded as statistically significant.

## Additional Information

**How to cite this article**: Lin, H. R. *et al*. Purification and differentiation of human adipose-derived stem cells by membrane filtration and membrane migration methods. *Sci. Rep.* **7**, 40069; doi: 10.1038/srep40069 (2017).

**Publisher's note:** Springer Nature remains neutral with regard to jurisdictional claims in published maps and institutional affiliations.
